# Neural oscillations and memory: unraveling the mechanisms of anesthesia-induced amnesia

**DOI:** 10.3389/fnins.2024.1492103

**Published:** 2024-11-14

**Authors:** Hui Liu, Zhanfei Yang, Yuxuan Chen, Fei Yang, Xue Cao, Gao Zhou, Yu Zhang

**Affiliations:** ^1^Department of Anesthesiology, Affiliated Hospital of Zunyi Medical University, Zunyi, China; ^2^Key Laboratory of Anesthesia and Organ Protection of Ministry of Education (In Cultivation), Zunyi Medical University, Zunyi, China; ^3^Guizhou Key Laboratory of Anesthesia and Organ Protection, Zunyi Medical University, Zunyi, China

**Keywords:** general anesthesia, neural oscillations, memory consolidation, synaptic plasticity, anesthesia-induced amnesia

## Abstract

General anesthesia is a widely used medical practice, affecting more than 300 million patients annually. Despite its ubiquity, the underlying mechanisms through which anesthetic agents induce amnesia remain poorly understood. This review explores the impact of general anesthetics on memory function, with a particular focus on the role of neural oscillations in anesthesia-induced memory suppression. Neural oscillations, such as theta, gamma, delta oscillations, slow oscillations (SO), spindles, and sharp wave ripples (SWR), are critical for memory formation and consolidation. Various anesthetics modulate these oscillations in ways that affect memory, even at subanesthetic concentrations. We highlight recent findings on the molecular and electrophysiological mechanisms by which general anesthetics influence memory-related neural oscillations, including the inhibition of synaptic plasticity, alterations in spike-timing-dependent plasticity (STDP), and disruption of cross-frequency couplings like theta-gamma and SO-spindle-SWR. Additionally, the review addresses the significance of age in anesthesia-related memory loss, with elderly patients being particularly vulnerable to long-term cognitive decline. Electrophysiological techniques, such as Electroencephalography (EEG); and advanced neuromodulation techniques, such as chemogenetics, and optogenetics, have provided insights into the neural dynamics underpinning anesthesia-induced amnesia, yet the causal relationship between EEG rhythms and memory impairment remains to be fully elucidated. This review underscores the importance of further research into the interaction between anesthesia, neural oscillations, and memory. Understanding these mechanisms will not only advance theoretical knowledge of general anesthesia but also aid in the development of safer anesthetic strategies to mitigate postoperative cognitive dysfunction, especially in high-risk populations.

## 1 Introduction

According to The Lancet, more than 300 million people worldwide undergo general anesthesia each year (Dobson, 2020). During general anesthesia, anesthetic agents induce central nervous system suppression, leading to amnesia. The amnestic effect is critical not only for inhibiting memory function during surgery to prevent intraoperative awareness but also for ensuring that this inhibition rapidly subsides postoperatively to avoid impairing the patient’s normal memory and recall abilities, which could result in postoperative cognitive dysfunction (POCD). Amnesia induced by general anesthetics usually refers to an inability to recall events or information occurring after the onset of anesthesia, known as anterograde amnesia. The mechanism underlying this amnesia is drug-induced interference with memory processes, with the degree of inhibition related to blood concentration levels, making it controllable, adjustable, and fully reversible. The precise mechanism of anesthesia-induced amnesia remains unclear, but it is currently believed to result from the disruption of new memory consolidation in the brain. Brain regions associated with memory include the hippocampus, cortex, amygdala, neostriatum, and cerebellum (Johnson, 2024).

Neural oscillations (electrophysiological markers) are rhythmic or repetitive patterns of neural activity generated in the central nervous system by large-scale synchronous activation of neuronal populations. This synchrony is crucial for inter-neuronal communication and information transfer (Doelling and Assaneo, 2021). These oscillations, observed in regions such as the hippocampus, thalamus, and cortex of mammals, play a vital role in complex cognitive processes like spatial navigation, memory, and learning (Akeju et al., 2014; Sharf et al., 2022). During surgery and general anesthesia, neural oscillations undergo changes depending on the dose of anesthetic agents. The underlying mechanisms likely depend on cellular characteristics such as ion currents, myelin integrity, and synaptic density (Akeju et al., 2014; Chamadia et al., 2019; Scheinin et al., 2018). Neural oscillations are essential for brain information processing, and oscillations induced by anesthetics may disrupt the brain’s ability to process information within cognitive, memory, and sensory pathways. Over the past few decades, significant progress has been made in understanding the mechanisms of general anesthesia at the molecular, cellular, and neural network levels (Hemmings Jr et al., 2019), but electrophysiological studies on anesthesia-induced memory suppression and amnesia remain scarce.

## 2 Molecular mechanisms of memory consolidation

Memory is first encoded in the hippocampus and gradually transferred to the cortex for long-term storage—a process known as memory consolidation (Goto et al., 2021). Synaptic plasticity, which refers to activity-dependent changes in the strength of neuronal connections, is a key mechanism in this process. Synaptic plasticity, particularly long-term potentiation (LTP) and long-term depression (LTD), has long been considered critical to learning and memory (Magee and Grienberger, 2020). Recent studies show that early stages of memory consolidation involve multiple synaptic plasticity steps, each of which may go through consolidation processes (Goto et al., 2021). This stepwise mechanism makes memory transfer and consolidation between brain regions more efficient and reliable. By selectively inhibiting hippocampal LTP, significant effects on memory formation and stability have been observed within specific time windows, highlighting the importance of these sequential plasticity events in memory consolidation (Goto et al., 2021). Synaptic transmission is a fundamental process for inter-regional information transfer in the central nervous system, and intact synaptic function is crucial for nearly all neural functions, including consciousness, memory, and cognition. A strong correlation exists between the inhibition of LTP by general anesthetics and memory impairment (Ishizeki et al., 2008; Hao et al., 2020).

The mechanism by which general anesthetics exert their effects primarily involves molecular targets, especially ion channels. These include ligand-gated ionotropic receptors such as *γ*-aminobutyric acid (GABA), glutamate, and acetylcholine neurotransmitter systems (Alkadhi, 2021), General anesthetics achieve their anesthetic effects by inhibiting excitatory synaptic transmission and enhancing inhibitory synaptic transmission through specific ion channels. By affecting synaptic transmission, they subsequently influence synaptic plasticity. For example, anesthetics may reduce the occurrence of LTP by inhibiting excitatory synaptic transmission, while enhancing inhibitory transmission may promote LTD. Synaptic plasticity relies on the dynamic changes in synaptic transmission, and neurotransmitter systems are key factors in regulating synaptic plasticity (Bruentgens et al., 2024). General anesthetics may impact memory by acting on specific molecular targets that affect synaptic plasticity. Recent studies have shown that repeated exposure to clinically relevant concentrations of sevoflurane can lead to neurodevelopmental dysfunction, eventually resulting in cognitive impairment. This process is accompanied by a reduction in postsynaptic density protein-95 (PSD-95), which has been used as a marker of synaptic plasticity. Therefore, sevoflurane’s effect on memory can be attributed to its impact on synaptic plasticity (Lu et al., 2017). Other general anesthetics also influence synaptic transmission through the regulation of specific ion channels and related targets (Platholi and Hemmings, 2022).

The induction of synaptic plasticity is coordinated by the precise timing of action potentials across neuronal populations, which can generate oscillations of different frequencies. Hippocampal synaptic plasticity (and hippocampal-dependent learning and memory) relies on the precise synchronization of neuronal firing with ongoing network activity (Rutishauser et al., 2010). Synaptic plasticity can be enhanced through STDP (Pang et al., 2019), a phenomenon where the strength of synaptic connections changes based on the precise timing between presynaptic and postsynaptic neuronal firing. If presynaptic neurons fire before postsynaptic neurons, synaptic enhancement typically occurs, resulting in LTP. In contrast, if presynaptic neurons fire after postsynaptic neurons, synaptic weakening or LTD occurs (Feldman, 2012; Song et al., 2000; Bi and Poo, 1998). This mechanism enables neuronal firing timing to determine the strengthening or weakening of synaptic connections, thus facilitating learning and memory formation (Debanne and Inglebert, 2023). Neural oscillations serve as a “pacemaker” in the nervous system, regulating inter-neuronal communication and shaping learning and memory processes through their influence on synaptic plasticity (Hanslmayr et al., 2024). These oscillations can affect synaptic plasticity through mechanisms such as specific frequency oscillations (Wong, 2022) and phase-locking effects (Wang D. et al., 2023). Studies also suggest that changes in synaptic activity can promote or interfere with the generation of neural oscillations. When synaptic transmission efficiency increases, stronger oscillatory patterns may emerge, influencing overall network activity. This feedback mechanism likely plays a critical role in information processing and storage (Craig et al., 2024). General anesthesia-induced amnesia may involve disruptions in hippocampal synaptic plasticity through altered neural oscillations.

**Figure 1:**
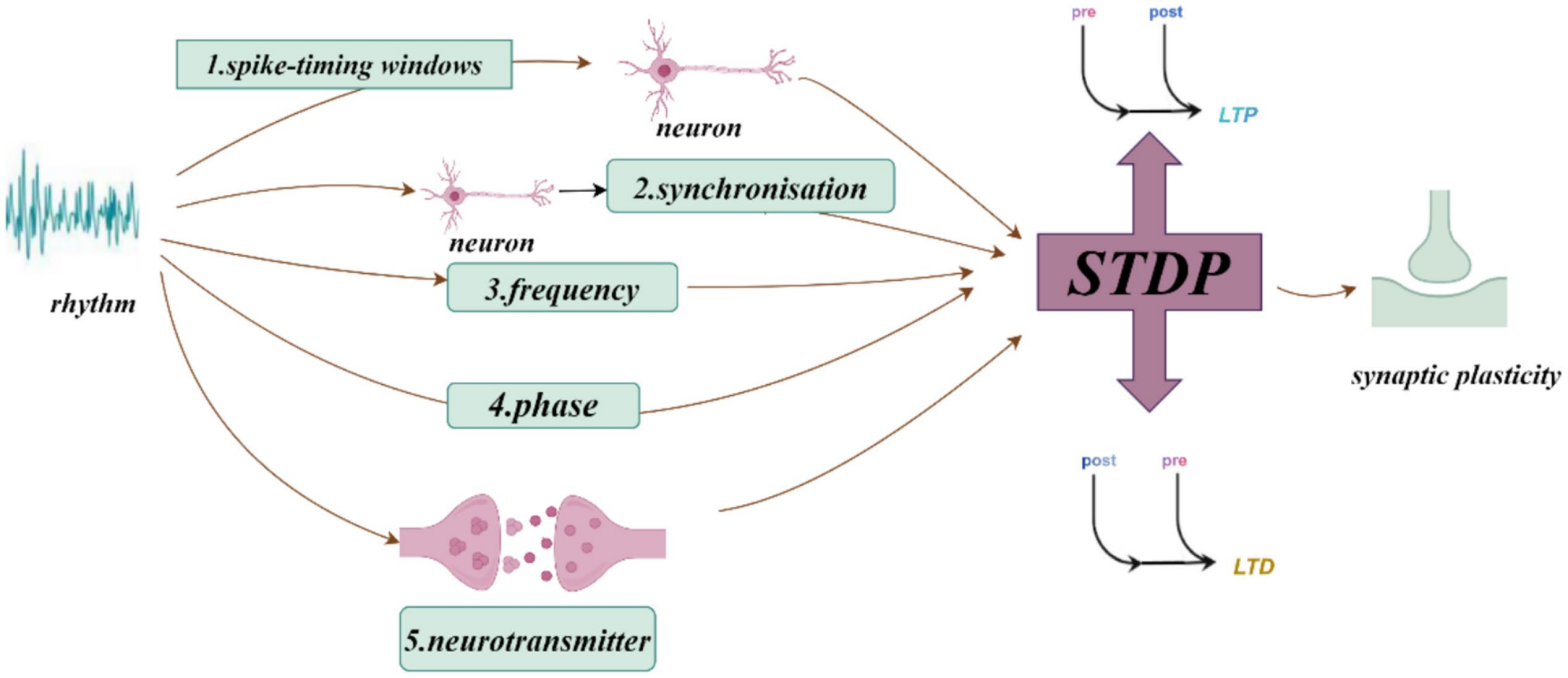
How neural oscillations influence synaptic plasticity and spike-timing-dependent plasticity (STDP).

**Figure 2:**
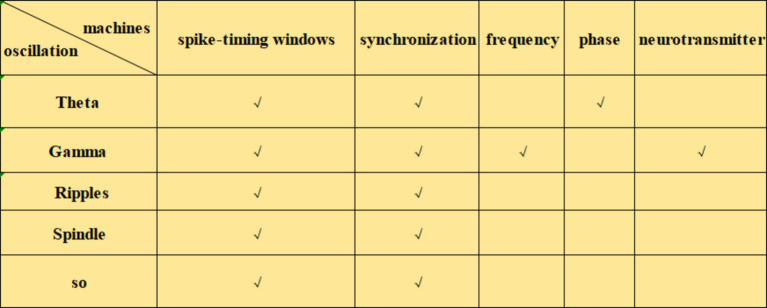
Mechanisms of the effects of different neural oscillations on STDP.

**Figure 3:**
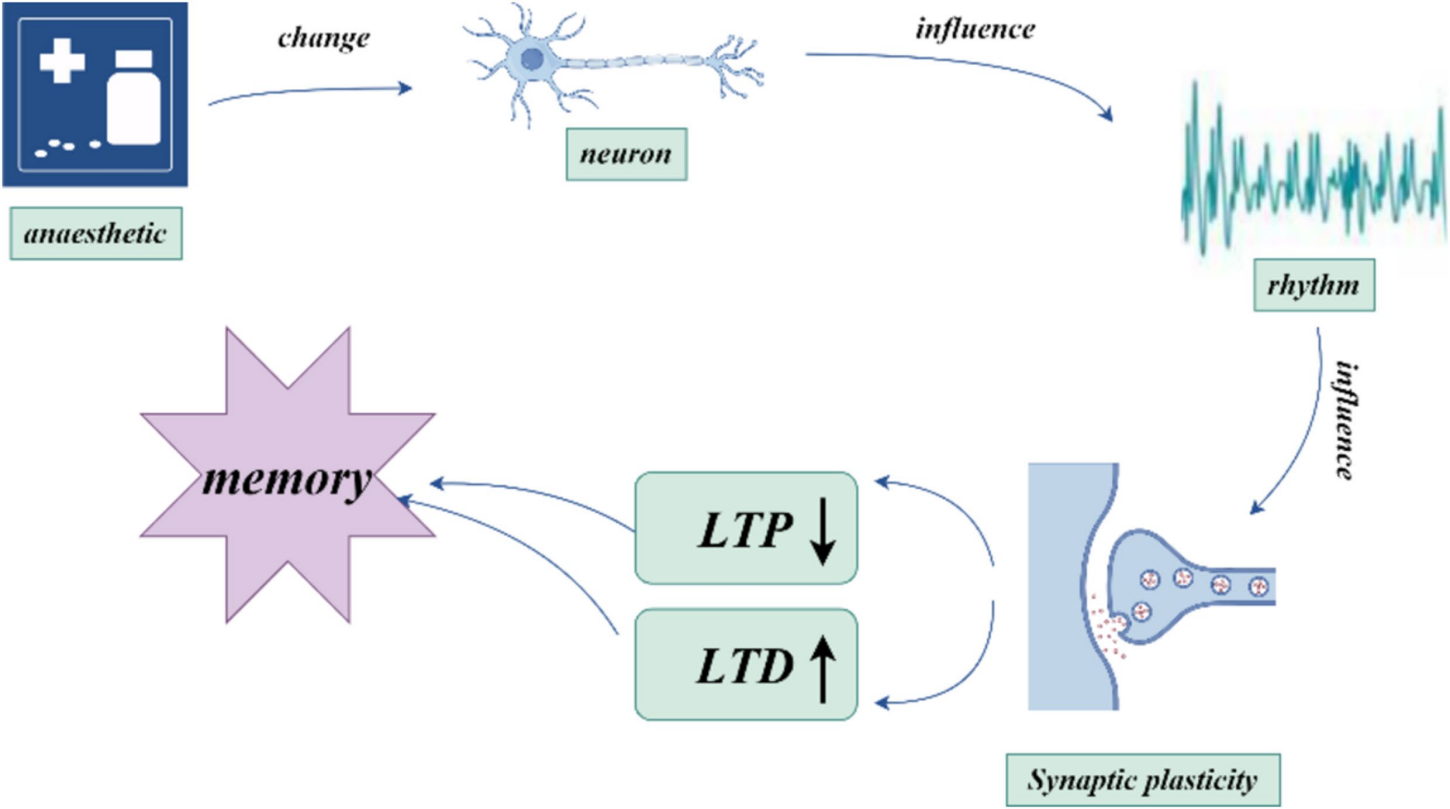
how anesthetics influence neural oscillations and memory.

Neural oscillations can influence synaptic plasticity by affecting STDP in several ways. (1) By modulating the timing of neuronal firing, neural oscillations create a specific time window for STDP. During the peak of neural oscillations, neuronal firing is likely to be more synchronized, which can enhance synaptic plasticity. Frequencies such as theta (Andrade-Talavera et al., 2023), gamma (Li et al., 2021; Griffiths and Jensen, 2023), ripple (Staresina, 2024), spindle (Dickey et al., 2021) and slow oscillations (SO) (Luz and Shamir, 2016) can coordinate pre-and postsynaptic firing within precise time frames, creating favorable conditions for STDP. This coordination facilitates synchronous firing among neurons, thus enhancing synaptic plasticity. The possible mechanisms by which neural oscillations affect STDP include: (2) Frequency dependence on STDP (Wong, 2022), particularly under high-frequency conditions such as gamma oscillations, which generate more action potentials within a short period and thus increase the likelihood of LTP (Wong, 2022). When neurons operate at gamma frequencies, their cooperative activity strengthens, directly influencing STDP efficacy and the manifestation of synaptic plasticity. (3) The phase of neural oscillations has a direct impact on STDP. Different phases of neural oscillations correspond to distinct time windows, where presynaptic firing during the rising phase of theta oscillations is more likely to induce LTP, while firing during the descending phase is more likely to induce LTD (Wang D. et al., 2023). (4) Neural oscillations can also regulate STDP by modulating neurotransmitter release. Changes in oscillatory frequency alter neurotransmitter release patterns, which in turn influence synaptic plasticity. For example, during gamma oscillations, neurotransmitters such as dopamine and glutamate may be released more synchronously, which enhances the effects of STDP (Lee et al., 2009). Theta stimulation, for instance, can modulate the release of dopamine neurotransmitters (Aceves-Serrano et al., 2022) (Figures 1–3).

## 3 Electrophysiological indicators related to memory

EEG is a reliable and valuable non-invasive diagnostic tool used to study the brain’s electrophysiological activity. It records changes in electrical potentials from the scalp, which reflect the bioelectric signals generated by neuronal communication. EEG oscillations can be classified into five types based on their frequency: alpha (*α*), beta (*β*), gamma (*γ*), delta (*δ*), and theta (*θ*) oscillations. Among these, theta, gamma, and delta oscillations are frequently reported in memory-related studies, along with slow oscillations, sleep spindles, and sharp wave ripples (SWRs). EEG is also a crucial tool for studying the effects of anesthesia on neural oscillations. By analyzing EEG data during anesthesia, researchers have discovered that neural oscillations at different frequencies vary according to the depth of anesthesia, providing important insights into the physiological mechanisms of anesthesia.

### 3.1 Theta oscillations

Theta oscillations, with a frequency of 4–12 Hz and an amplitude of 100–150 μV, are one of the most prominent and sinusoidal brain activity patterns (Colgin, 2013). The hippocampus receives major excitatory input from the entorhinal cortex and widespread cholinergic and GABAergic input from the medial septum. During active exploration, these three regions exhibit synchronized rhythmic activity, known as theta oscillations (Buzsáki, 2005). Theta oscillations generated in hippocampal and cortical neuronal networks are involved in various brain functions, including sensorimotor integration, motor planning, memory formation, and attention. The generation of theta rhythms involves specific interactions between cellular (ion channels) and network (synaptic) mechanisms (Colgin, 2013).

One study used light and sound stimulation to synchronize brain oscillations to a target frequency, specifically theta oscillations. The participants who underwent this brainwave synchronization training performed significantly better in memory tests compared to the untrained group, suggesting that enhancing theta oscillations positively affects memory improvement (Addante et al., 2021). In a traumatic brain injury mouse model, optogenetic stimulation of hippocampal CA1 pyramidal neurons at theta frequency enhanced memory performance during a recognition memory task (Broussard et al., 2023). Additionally, selective silencing of medial septal GABAergic neurons (MS (GABA)) during rapid eye movement (REM) sleep, which precisely attenuates theta rhythms without disrupting sleep behavior, significantly impaired contextual fear memory (Boyce et al., 2016). These findings indicate that theta rhythms are closely associated with memory function.

Typically, theta rhythms are associated with the enhancement of learning and memory. However, recent studies have shown that in certain circumstances, particularly under the influence of anesthetics like sevoflurane, an increase in theta rhythm may have a negative impact on memory. Clinical studies have shown that various inhaled anesthetics (isoflurane, sevoflurane, halothane, and desflurane) significantly enhance the intensity of theta rhythm oscillations in the prefrontal cortex at surgical anesthesia concentrations (Akeju et al., 2014). Sevoflurane has been found to cause memory impairment during clinical anesthesia, though the underlying mechanisms remain largely unknown. The TASK-3 channel is one potential target of sevoflurane. It enhances theta rhythms in the hippocampus, which are associated with memory. Virus-mediated knockout of the hippocampal TASK-3 channel significantly reduced sevoflurane-induced enhancement of theta rhythms and mitigated memory impairment, suggesting that sevoflurane may impair memory by increasing hippocampal theta oscillations through the TASK-3 channel (Han et al., 2021). Additionally, studies on three inhaled anesthetics (halothane, nitrous oxide, and isoflurane) at subanesthetic concentrations in freely behaving rats showed that all three anesthetics slowed the peak frequency of theta oscillations. The decrease in theta peak frequency was dose-dependent and correlated with the suppression of memory-related fear conditioning behaviors (Perouansky et al., 2010). This suggests that the modulation of hippocampal theta oscillations by inhaled anesthetics may be involved in the mechanisms of anesthesia-induced amnesia. The role of theta rhythms is not fixed, and it can exhibit entirely different effects under various physiological and pharmacological conditions. Further research will help uncover these mechanisms and provide theoretical support for improving clinical anesthesia practices.

### 3.2 Gamma oscillations

Gamma oscillations, with a frequency range of 20–100 Hz, are commonly observed as synchronous neural activity in various brain regions such as the olfactory bulb, thalamus, hippocampus, and sensory and motor cortices. They are closely associated with normal cognitive functions, such as attention, memory, and learning, particularly in the hippocampus (Guan et al., 2022). Gamma oscillations contribute to successful episodic memory formation and retrieval through three neural mechanisms: spike-timing-dependent plasticity, neural communication, and sequence encoding/retrieval (Griffiths and Jensen, 2023). Gamma oscillations are further subdivided into low-frequency (30–60 Hz) and high-frequency (60–120 Hz) bands, which are believed to have different functional roles. High-frequency gamma oscillations are closely linked to cognitive task performance, playing an important role in memory, attention, and perception, especially during complex cognitive tasks. For instance, high-frequency gamma oscillations in the prefrontal cortex (mPFC) are often observed just before visual stimuli appear (Dubey and Ray, 2020). In contrast, low-frequency gamma oscillations are more often associated with basic neural functions and states.

Research has shown that optogenetic gamma stimulation can rescue memory deficits in mouse models of Alzheimer’s disease (Etter et al., 2019). Specifically, 40 Hz optogenetic stimulation was found to increase functional synaptic plasticity (Wang C. et al., 2023). Isoflurane, however, has been shown to reduce high-frequency gamma oscillations, while low-frequency gamma oscillations remain unaffected (Hudetz et al., 2011). Propofol, sevoflurane and ketamine decrease the power of high-frequency gamma oscillations (Pal et al., 2017). Dexmedetomidine is a sedative drug that induces an increase in the power of gamma oscillations during general anesthesia in the moderate mode of sedation, but the power of gamma oscillations decreases in the deep mode of sedation (Xi et al., 2018). Surgery and anesthesia can disrupt normal gamma oscillations in the brain through pathological mechanisms such as neuroinflammation (Ji et al., 2020), sleep disturbances (Tabassum et al., 2021), amyloid-beta (Aβ) deposition (Chung et al., 2020), and tau hyperphosphorylation (Richetin et al., 2020), leading to perioperative neurocognitive disorders. The amnestic effects of anesthesia may also be related to its impact on gamma oscillations.

### 3.3 Slow wave oscillations

Cortical slow wave activity is a key marker of reduced wakefulness and is a rhythmic neocortical activity that occurs during non-rapid eye movement (NREM) sleep. These oscillations have a frequency of 0.5–4.0 Hz and are generated primarily by excitatory and inhibitory postsynaptic potentials from pyramidal neurons in cortical layers III, V, and VI (Feyissa and Tatum, 2019). Increasing evidence shows that slow wave activity is closely related to neuroplasticity, central nervous system development, and cognitive function, and in basic anesthesia research, the proportion of SO is commonly used to assess anesthetic effects. Slow wave activity includes both SO and delta oscillations. During slow-wave sleep (SWS) and anesthesia, SO typically occur at frequencies below 1 Hz and are referred to as SO. Delta oscillations, typically within the 1–4 Hz range, are considered the result of cortical silencing during rest. Delta oscillations have two primary origins: one from the cortex, the mechanism of which is unclear but may be related to cognitive function in the waking state; and the other from the thalamus, which is better understood and closely associated with sleep.

SO and delta oscillations during sleep have been increasingly linked to memory consolidation. A French study showed that delta oscillations, which were previously thought to alternate with active states during SWS and represent moments of cortical silence, are actually involved in the formation of long-term memory. The researchers found that delta oscillations in the brain during sleep do not become silent with cortical inactivity but instead isolate clusters of neurons, aiding long-term memory formation. By artificially inducing delta oscillations in a rat model to isolate neurons related to hippocampal reactivation, memory was consolidated. Delta oscillations are thus seen as a method of selectively isolating specific neuronal groups that transmit critical signals between the hippocampus and cortex to form long-term memory (Todorova and Zugaro, 2019).

Common anesthetics, such as propofol and sevoflurane, induce stronger and slower delta oscillations as doses increase and consciousness diminishes (Adam et al., 2023). The question of whether memory loss during unconsciousness is related to changes in brain rhythms remains to be explored. Crick & Koch proposed that the claustrum coordinates activity across most neocortical areas, regulating the generation of neocortical SO. A recent study on claustrum regulation of propofol anesthesia EEG activity found that propofol reduces slow wave activity by inhibiting neurons and their associated receptors in the claustrum (Luo et al., 2023). Ketamine, another general anesthetic, reduces the effects of weak tonic activation of thalamic reticular nucleus (TRN) neurons on producing SO in local ipsilateral cortical areas, thereby inducing reduced delta oscillations (Mahdavi et al., 2020). Whether anesthetic-induced changes in SO can affect memory requires further investigation.

### 3.4 Sharp wave ripples

Sharp wave ripples (SWRs) are a complex of two distinct events: ripples of 110–250 Hz are superimposed on sharp waves of 0.1–3 Hz. The sharp wave reflects strong synchronous depolarization of pyramidal neuron dendrites, while high-frequency ripple waves reflect interactions between pyramidal neurons and their surrounding interneurons. Although SWRs are composite events, the sharp wave and ripple components are functionally distinct: the sharp wave is an excitatory potential transmitted from the hippocampal CA3 to the CA1 subregion, while the ripple is generated by the spiking of basket cells in the CA1 region (Schlingloff et al., 2014; Zhou and Norimoto, 2023). SWRs are most commonly observed during slow-wave sleep, immobilization, and exploration of new environments and are associated with widespread brain activity changes. Ripples are especially frequent during NREM sleep, though they can also be detected during wakefulness.

SWRs are believed to play an important role in memory consolidation. According to the “two-stage” model of memory consolidation, during the learning phase, the cortex provides new information to the hippocampus, facilitating rapid and temporary reorganization of hippocampal circuits. During relaxation or slow-wave sleep, spontaneously generated sharp waves and ripples in the brain continually retransmit the reorganized or integrated information from the hippocampus to the cortex for long-term storage (Lewis and Durrant, 2011). Animal studies have shown that eliminating ripples in the hippocampus during the post-learning memory consolidation stage significantly impairs spatial memory in rats (Girardeau et al., 2009). Optogenetic activation of spontaneous ripple oscillations in the hippocampal CA1 region prolonged ripple events and significantly improved performance on hippocampus-dependent memory tasks in rats, whereas halting ripple activity did not increase memory performance, and longer ripple events were observed when memory demands were high (Fernández-Ruiz et al., 2019).

The amnestic effects of general anesthetics may be linked to their impact on hippocampal ripple oscillations. Early research demonstrated that pentobarbital could inhibit or even eliminate ripple oscillations (Suzuki and Smith, 1988). Thiopental, at memory-inhibiting concentrations, significantly suppressed the occurrence and duration of ripple oscillations *in vitro* (Papatheodoropoulos et al., 2007). Benzodiazepines such as diazepam have been shown to reduce ripple oscillation frequency and amplitude, while the benzodiazepine antagonist flumazenil can reduce the occurrence, amplitude, and duration of ripple oscillations (Ponomarenko et al., 2004). Isoflurane at subanesthetic concentrations mediates effects on hippocampal ripple oscillations through differential modulation of pyramidal neuron and interneuron excitability, potentially contributing to its amnestic effects by lowering ripple frequency and aiding in memory clearance (Zhao et al., 2021). In mice anesthetized with urethane, *in vivo* patch-clamp recordings from dorsal hippocampal CA1 neurons revealed that urethane anesthesia reduced membrane potential fluctuations, decreased synchronous spikes in hippocampal neurons, and lowered hippocampal sharp wave ripple amplitude, reflecting reduced synaptic activity. Compared to natural rest or sleep conditions, the memory consolidation mechanism in the hippocampus, supported by neuronal spike synchrony, may be weakened under urethane anesthesia (Yagishita et al., 2020).

### 3.5 Sleep spindles

Spindles refer to a readily identifiable sequence of 10–15 Hz sinusoidal cycles in the EEG of sleeping mammals, usually occurring during stage N2 of NREM sleep (Fernandez and Lüthi, 2020). GABAergic inhibitory neurons in the thalamic reticular nucleus regulate thalamocortical neuronal activity to generate spindles. The function of spindles includes protecting sleep by inhibiting sensory input and promoting the formation of learning and memory abilities (Fernandez and Lüthi, 2020). Changes in NREM oscillations after learning were first demonstrated in human studies, where specific spindle parameters (such as density and amplitude) were found to increase during post-learning sleep in declarative and procedural memory tasks, reflecting the strength and plasticity of thalamocortical circuits, as well as large-scale functional connectivity and plasticity (Clemens et al., 2005; Chen et al., 2024). Rosanova et al. demonstrated a direct link between spindles and synaptic plasticity *in vitro*. They showed that natural spindle stimulation from *in vivo* intracellular recordings induced short-term potentiation (STP) and LTP in somatosensory cortical slices (Rosanova and Ulrich, 2005). In humans, combining neurostimulation with paired associative stimulation (PAS) for LTP-or LTD-like induction revealed that increases or decreases in slow spindle activity correlated significantly with the effects of PAS-induced LTP or LTD (Peyrache and Seibt, 2020; Bergmann et al., 2008).

**Figure 4:**
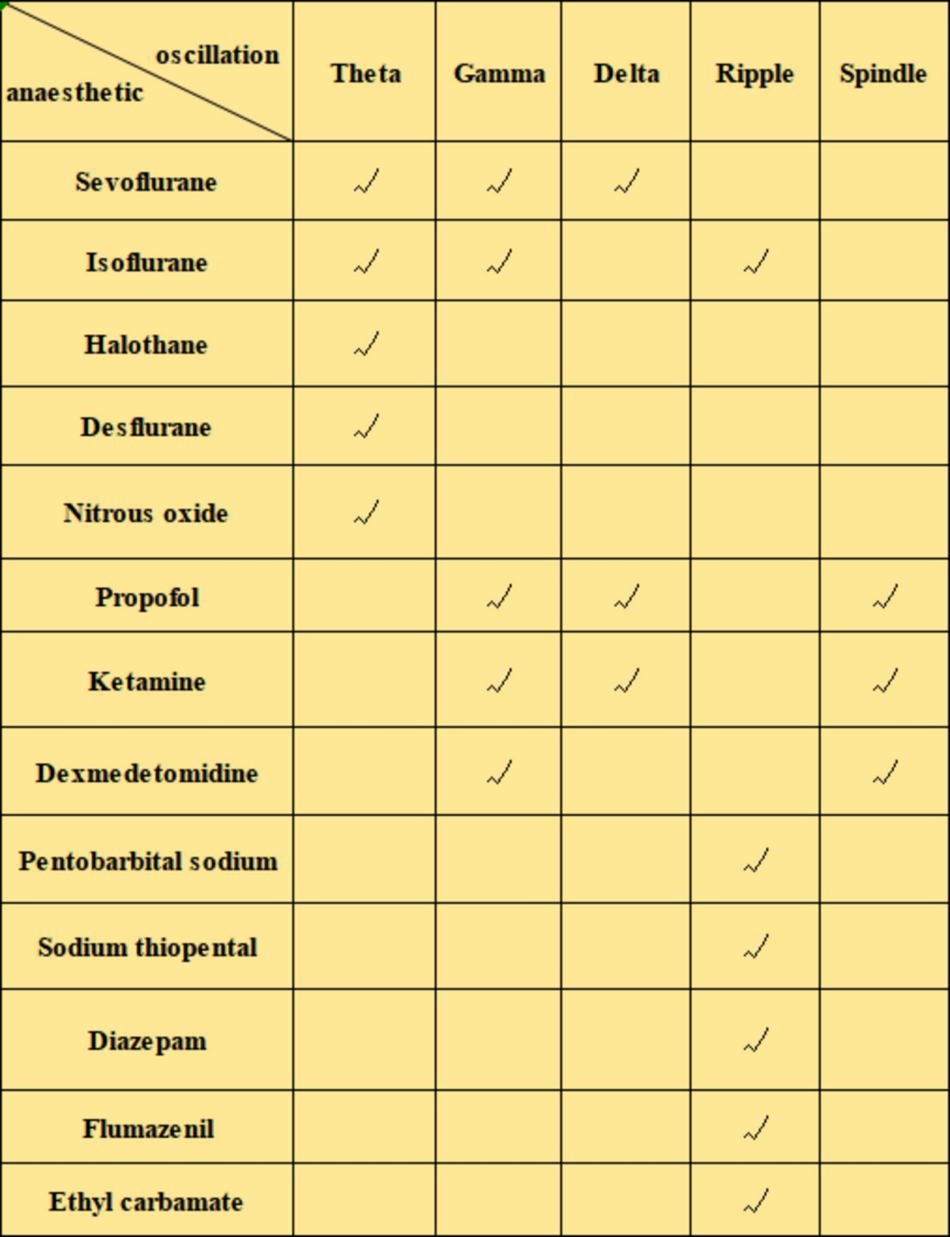
Effects of different anesthetics on nerve oscillations.

Early studies using zolpidem (a GABA agonist) to enhance sleep spindles during daytime naps found that it promoted hippocampus-dependent episodic memory (Zhang et al., 2020). Non-benzodiazepine hypnotics like eszopiclone act on GABAergic neurons in the thalamic reticular nucleus, which generates spindles and increases the association between spindles and motor learning (Wamsley et al., 2013). General anesthetics such as ketamine can switch thalamic reticular neuron firing from burst to tonic mode, thereby reducing spindles in the thalamocortical system (Mahdavi et al., 2020). The intravenous anesthetics propofol and dexmedetomidine induce an increase in spindle activity (Xi et al., 2018). While spindles are known to enhance memory consolidation, the presence of certain anesthetics can lead to increased spindle activity but may negatively impact memory. This negative effect is likely due to the inhibitory influence of anesthesia on neuronal activity, which can disrupt the integration and consolidation of information. The relationship between spindle generation during anesthesia and amnesia is an area worth exploring (Figure 4).

## 4 Interaction of different frequency oscillations and their effects on memory

In addition to individual frequency bands, the interaction between different oscillatory bands plays a crucial role in working memory. Lower frequency oscillations (theta, alpha, beta) often co-occur with higher frequency gamma oscillations, where high-frequency oscillations represent neuronal ensemble encoding of external information, and lower frequency oscillations reflect interregional information transfer. The specific role of these oscillatory couplings in working memory can be examined by modulating the interactions between different frequency bands (Arski et al., 2020; Daume et al., 2017).

### 4.1 Theta-gamma coupling

Theta-gamma coupling (TGC) is a neurophysiological mechanism supporting working memory (WM), in which the interaction between two oscillations of different frequencies is referred to as cross-frequency coupling. In phase-amplitude coupling, the amplitude of high-frequency oscillations is modulated by the phase of low-frequency oscillations (Salimpour et al., 2022). One example of this is theta-gamma coupling, where the phase of theta oscillations (4–8 Hz) modulates the amplitude of gamma oscillations (30–80 Hz). Animal models suggest that gamma oscillations represent individual items to be remembered, while theta oscillations represent the time intervals for these items, with modulation of gamma amplitude by theta phase encoding the sequential order of the presented information (Brooks et al., 2022).

In one study, ultrasound stimulation was applied to the hippocampal CA1 region of anesthetized and awake mice, with simultaneous local field potential recordings. The results showed a significant increase in absolute theta and gamma power following ultrasound stimulation in both anesthetized and awake states. Under anesthesia, increasing ultrasound intensity led to a decrease in relative theta power and an increase in gamma power, whereas the opposite effect was observed in the awake state (Li et al., 2023). Thus, it is worth exploring whether the amnestic effects of anesthesia are related to theta-gamma coupling.

### 4.2 SO-spindle-delta oscillation coupling

Sleep is critical for balancing learning and forgetting, reinforcing some memories while erasing others through different brain electrical patterns. SOs and delta oscillations are rhythmic neuronal activities that serve as markers of sleep, but their specific functional roles are difficult to distinguish. For decades, research on memory enhancement and loss has focused primarily on two brain wave patterns: SOs and spindles. When SOs are coupled with spindles, they become essential for memory consolidation. SOs and delta oscillations often co-occur during sleep and are difficult to differentiate, commonly classified together as slow oscillations. Using closed-loop optogenetics in rats, SOs were separated from delta oscillations, revealing that SOs aid in memory consolidation, while delta oscillations contribute to forgetting (Kim et al., 2019).

### 4.3 SO-spindle-SWR coupling

A substantial body of literature demonstrates that SOs (It reflects the overall fluctuations in excitability across large populations of neurons), spindles, and SWRs play three distinct roles in memory consolidation. All three rhythms are involved in behavioral and physiological memory-related processes, yet they appear to serve different functions. These oscillations are the dominant rhythms during stage N2 of NREM sleep. Spindles promote structural changes in the brain and display greater spatial variability, tending to express at locations involved in prior learning and shaping relevant memory networks. Finally, SWRs have the most direct influence on neuronal firing rates and communication, driving plasticity within and between local target circuits. Numerous studies in rodents and humans have shown that these three sleep rhythms are temporally coupled (Oyanedel et al., 2020).

The coupling of SOs, spindles, and SWRs regulates neuronal firing rates and communication, continuously enhancing their efficiency and spatiotemporal precision to serve learning and plasticity. This coordinated coupling controls the consolidation process with increasing precision, facilitating the reactivation, refinement, and redistribution of memory traces across hippocampal-cortical networks (Staresina, 2024). General anesthesia alters these oscillations individually, but their coupling and the impact on anesthesia-induced amnesia require further exploration.

## 5 Discussion

Different EEG rhythms have varying impacts on memory function, and different anesthetics affect EEG rhythms differently, potentially playing a role in anesthesia-induced amnesia. Talkie et al. discovered that even at subanesthetic concentrations (0.25%), sevoflurane can impair the formation of emotional memory in humans, with dose-dependent suppression effects. This suggests that the concentration of anesthetics required to inhibit memory is lower than that needed to suppress consciousness (Alkire et al., 2008). Neural oscillations, which are rhythmic fluctuations in neuronal electrical activity, are closely associated with memory. The loss of consciousness induced by general anesthetics leads to significant alterations in neural oscillations, and subanesthetic states can already result in memory impairment. Our clinical anesthesia practice typically uses concentrations that induce unconsciousness, which have an even stronger impact on memory. Therefore, analyzing neural oscillations during general anesthesia may help unravel the mystery of anesthesia-induced amnesia. The effects of anesthesia on neural oscillations are complex and significant, and future research utilizing advanced neuroimaging and electrophysiological recording techniques will further elucidate the impact of anesthesia on neural oscillations. This will enhance our understanding of anesthetic mechanisms and their clinical applications.

Research has shown that age is a significant factor influencing memory loss after anesthesia. Elderly patients face a higher risk of long-term memory loss following anesthesia (Amiri et al., 2020). While short-term memory loss is a common phenomenon after anesthesia, the impact is more pronounced in elderly individuals and those at risk for cognitive impairment. Future studies must explore the long-term effects of anesthesia on the brain and develop effective strategies for managing and preventing postoperative memory loss. Continued research in this area holds promise for identifying safer anesthetic techniques that minimize the risk of POCD and postoperative delirium (POD), particularly in elderly patients.

Advancements in surgery and anesthesia can improve the function and quality of life for elderly patients, but they also present potential risks to brain health. Research shows that after surgery, particularly in elderly patients, there is a higher incidence of POCD. It is estimated that up to 65% of patients aged 65 and older experience delirium, and 10% may develop long-term cognitive decline (Mahanna-Gabrielli et al., 2019). POCD refers to a decline in memory, attention, and cognitive abilities following surgery. POD is an acute cognitive impairment that typically manifests as difficulties in attention, reduced cognitive function, and altered levels of consciousness. Surgeons and anesthesiologists should assess, discuss, and optimize the potential risks for each patient before surgery. Different types and dosages of anesthetics may have varying effects on cognitive function. The current research on the electrophysiological mechanisms of anesthesia-induced amnesia provides theoretical support for improving clinical anesthesia practices. By selecting anesthetics and protocols tailored to individual patients and adopting more refined anesthesia management strategies, the impact on cognitive function can be minimized, reducing the incidence of POCD and POD. The neural oscillations associated with anesthesia-induced amnesia, as discussed in this paper, can be detected via EEG. A meta-analysis on EEG monitoring and POD suggests that patients monitored with EEG may have a lower incidence of postoperative delirium (Mackenzie et al., 2018). EEG monitoring can also help healthcare providers identify early signs of delirium, allowing for more effective interventions.

## 6 Conclusion and outlook

The mechanism of general anesthesia remains one of the most important unresolved scientific problems worldwide. Investigating the impact of anesthetics on learning and memory functions will provide a theoretical foundation for further understanding the mechanisms of general anesthesia. Additionally, research in this area will contribute to the development of precise clinical anesthesia management and may provide valuable insights for the treatment of anesthesia-induced memory impairment, carrying profound clinical and scientific significance. As research into the mechanisms of general anesthesia deepens, more attention must be paid to the amnestic effects of anesthetics. General anesthetics can mediate amnesia by acting on specific electrophysiological mechanisms and targets. A deeper understanding of how anesthetics influence memory will enhance our ability to study memory processes. To date, perioperative EEG biomarkers of memory function are scarce, and the impact of different EEG markers on anesthesia-induced amnesia remains unclear. Techniques such as EEG and *in vivo*/*in vitro* electrophysiological techniques like whole-cell patch-clamp and advanced neuromodulation techniques, such as chemogenetics, and optogenetics, recording can be employed to analyze the electrophysiological mechanisms of various neural oscillations during anesthetic-induced amnesia. The extent to which neural oscillations contribute to anesthesia-induced amnesia, and whether there is a causal relationship between EEG rhythms and amnesia, requires further investigation.
